# Supporting strategic health purchasing: a case study of annual health budgets from general tax revenue and social health insurance in Abia state, Nigeria

**DOI:** 10.1186/s13561-021-00346-8

**Published:** 2021-12-20

**Authors:** Chinyere Mbachu, Chinyere Okeke, Chinonso Obayi, Agnes Gatome-Munyua, Nkechi Olalere, Ikechi Ogbonna, Benjamin Uzochukwu, Obinna Onwujekwe

**Affiliations:** 1 Health Policy Research Group, College of Medicine University of Nigeria, Ituku-Ozalla, Enugu, Nigeria; 2 Department of Community Medicine, College of Medicine University of Nigeria, Ituku-Ozalla, Enugu, Nigeria; 3 Strategic Purchasing Africa Resource Center (SPARC), Nairobi, Kenya; 4 Results for Development (R4D), Nairobi, Kenya; 5 Abia State Ministry of Health, Umuahia, Nigeria; 6 Department of Health Administration and Management, College of Medicine University of Nigeria, Ituku-Ozalla, Enugu, Nigeria

**Keywords:** Strategic health purchasing, SHP, Social health insurance, Annual budgets

## Abstract

**Background:**

Tracking general trends in strategic purchasing of health financing mechanisms will highlight where country demands may exist for technical support and where progress in being made that offer opportunities for regional learning. Health services in Abia State, Nigeria are funded from general tax-revenues (GTR), and a new state social health insurance scheme (SSHIS) is proposed to overcome the failings of the GTR and expand coverage of services. This study examined purchasing functions within the GTR and the proposed SSHIS to determine if the failings in GTR have been overcome, identify factors that shape health purchasing at sub-national levels, and provide lessons for other states in Nigeria pursuing a similar intervention.

**Methods:**

Data was collected through document review and key informant interviews. Government documents were retrieved electronically from the websites of different organizations. Hard copies of paper-only files were retrieved from relevant government agencies and departments. Interviews were conducted with seven key personnel of the State Ministry of Health and State Health Insurance Agency. Thematic analysis of data was based on a strategic health purchasing progress tracking framework which delves into the governance arrangements and information architecture needed for purchasing to work well; and the core purchasing decisions of what to buy; who to buy from; and how to buy.

**Results:**

There are differences in the purchasing arrangements of the two schemes. Purchaser-provider split does not exist for the GTR, unlike in the proposed SSHIS. There are no data systems for monitoring provider performance in the GTR-funded system, unlike in the SSHIS. Whereas GTR is based on a historical budgeting system, the SSHIS proposes to use a defined benefit package, which ensures value-for-money, as the basis for resource allocation. The GTR lacks private sector engagement, provider accreditation and contracting arrangements while the SSHIS will accredit and engage private providers through selective contracting. Likewise, provider payment is not linked to performance or adherence to established standards in the GTR, whereas provider payment will be linked to performance in the SSHIS.

**Conclusions:**

The State Social Health Insurance has been designed to overcome many of the limitations of the budgetary allocation to health. This study provides insights into the enabling and constraining factors that can be used to develop interventions intended to strengthen the strategic health purchasing in the study area, and lessons for the other Nigeria states with similar characteristics and approaches.

**Supplementary Information:**

The online version contains supplementary material available at 10.1186/s13561-021-00346-8.

## Background

Health purchasing is one of the three health financing functions, with the other two functions being funds mobilization and pooling. Hence, it involves the allocation or transfer of resources (pooled funds) to service providers in order to deliver healthcare goods and services to the population based on the defined benefit package [1]. The purchaser may be the Ministry of Health, an insurance scheme or an autonomous agency. Evidence shows that public expenditure on health is poor in Nigeria and healthcare purchasing has been predominantly passive and inefficient [2, 3], partly attributed to limited understanding and appreciation for strategic health purchasing.

Purchasing can be passive or strategic, although approaches to purchasing can be a continuous process from more passive to more strategic purchasing [4, 5]. In passive purchasing, purchasers transfer funds to service providers without due diligence on either the performance of the service providers or need for such health services to the population, quality and cost of delivering those services [3]. Funds are transferred to service providers based on predetermined budgets without considering efficiency [4]. As countries explore ways to increase the efficiency of limited resources to achieve the maximum health outcomes possible, the demand for Strategic Health Purchasing (SHP) has increased. SHP is defined as *the* efficient allocation of pooled funds to healthcare providers for the delivery of health services on behalf of a population. It involves three key decisions made by a purchaser about what to buy, from whom to buy and how to buy [6]. These decisions are at the core of making better decisions on resource allocation, creating better incentives and holding decision-makers accountable for effective health spending [6–8]. Hence, the objectives of strategic purchasing are to enhance equity in the distribution of resources, increase efficiency in the use of these resources, manage expenditure growth and promote quality in health service delivery with hopes to accelerate progress towards universal health coverage [6]. SHP across various health financing mechanisms is a tool for achieving universal health coverage (UHC). Success stories have been reported in low and middle income countries including Indonesia, Thailand and Cambodia [1, 9–13].

Recent health financing reforms in Nigeria such as the Basic Healthcare Provision Fund (BHCPF) and state-supported social health insurance schemes recognize the critical roles of state governments and related agencies in defining, deciding, implementing and managing healthcare purchasing functions within decentralized health financing mechanisms, as a means of fast-tracking the country’s achievements of UHC by 2030 [14]. To this end, States have been encouraged to establish social health insurance schemes to increase access to health services and reduce high out-of-pocket spending estimated at 76% of total health expenditure. Through the BHCPF, States are funded to set up these social health insurance schemes, and channel BHCPF subsidies to cover priority health services. Abia State in Nigeria has proposed to set up a State Social Health Insurance Scheme (SSHIS) for coverage to all residents of the State. The SSHIS design was intended to overcome the shortcomings of the annual budgets funded through general tax revenue by creating a purchaser-provider split, contracting of providers, using output based provider payment methods, and improving quality assurance.

Although evidence on health purchasing and strategic purchasing in Africa is growing the pool of literature on health purchasing arrangements in Africa remains underdeveloped. In Nigeria, the effects of multiple funding flows to providers have been studied and the operations of specific schemes, however there lacks literature on how these schemes interact, what is working and not working to provide recommendations for decision makers on how better to align health financing schemes to achieve UHC goals. Moreover, there is limited knowledge of factors that shape purchasing in health financing schemes at sub-national level in low-resource countries. Specific to Abia state, there is a lack of studies that provide a State level view of the health financing arrangements and details of how purchasing works.

This study examined the purchasing arrangements of the tax-funded annual budgetary allocation to health and the proposed state social health insurance scheme in Abia State Nigeria to assess whether and how these functions support SHP. The study also identifies where there are gaps and recommends strategies for moving towards SHP in the state. This information would be useful to policymakers and programmes to enhance SHP within tax-funded budgetary allocations to health and social health insurance systems.

## Methods

### Description of study area

Abia State is located in the Southeast region of Nigeria and covers approximately 5.8% of the total land area of Nigeria [15]. The State has a 2019 projection population of 4.5 million [16]. The State is endowed with natural resources and vast amounts of arable land and water sources and has a gross domestic product (GDP) per capita of $1799.25 [17]. About 70% of the population are involved in agriculture which contributes about 27% of the GDP [16]. The positive social determinants of health in the State include: high literacy rate (98.2% female; 94.2% men); 83.5% of households with improved source of drinking water; 76.5% of households with improved sanitary facilities; 81.7% of households with electricity; and employment rates of 59.7% among females and 74.4% among males [16]. Abia State is experiencing a rapid epidemiological and demographic transition from communicable diseases to non-communicable diseases (NCDs) [18].

Health service delivery is structured along 3 levels of care namely: primary, secondary and tertiary. There are 2 tertiary hospitals, 15 general hospitals located across the 17 LGA’s, and a total of 687 public primary health care centers distributed across political wards [19]. There are also 236 private hospitals and health facilities located mostly in semi-urban and urban areas [20], and eleven (11) training institutions for various cadres of health workers exist [19]. The health system is characterized by both state and non-state actors with defined responsibilities across various components of the health system. Collaborations exist between the government, private sector and partners in health care service delivery. Most health programmes are designed to allow for the integration of the various health actors in the implementation process at various levels. The State Ministry of Health and its line agencies regulate and coordinate health activities in the state.

The National Health Accounts of 2016 reported total health expenditure (THE) and THE per capita of $327.1 and $72, respectively, for Abia State [21]. In 2017, health was targeted as one of the 5-pillar Development Initiatives by the state government and a budgetary allocation of ₦4,421,943,000 (38.9% of the Capital Allocation) was made to the health sector [22]. Likewise, the state was one of the first to pay the counterpart funds for the Basic Health Care Provision Fund, and 186 PHCs have received the first tranche of payments for implementation of a basic minimum package of health services in line with the BHCPF operational guideline [23]. The state has established its Health Insurance Scheme governed by Abia State Health Insurance Agency (ABSHIA) to improve financial access to health for citizens. Enrolment has also begun for public sector employees only, with plans underway to expand to the private sector.

### Conceptual and analytic framework

**Fig. 1:**
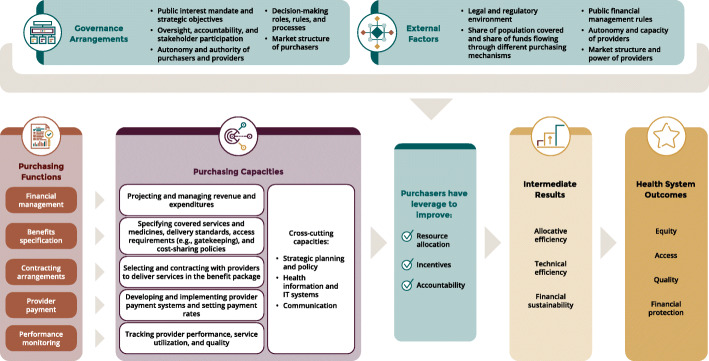
SHP progress monitoring framework (*Source: Strategic Purchasing Africa Resource Center*)

This paper adopted the SHP progress tracking framework which was co-developed by the Strategic Purchasing Africa Resource Center (SPARC) and the consortium of technical partners (Fig. 1) (Cashin C, Gatome-Munyua A, Kiendrébéogo JA: A Functional approach to making progress on strategic health purchasing for universal health coverage, forthcoming). The framework draws upon existing guidance documents and empirical evidence of SHP in LMICs and high-income countries alike [24]. The SHP progress framework was chosen for this study because it enables understanding of the critical functions of SHP and how governments and other stakeholders can improve their capacity to fulfil critical SHP functions or design and adapt SHP reforms. The structure and components of the framework that concern the purchasing system are summarized in Table 1.

**Table 1:** Purchasing Functions Needed to Support the SHP System

Governance arrangements & information architecture	- Legal, regulatory and governance structures (including stakeholder groups and accountability_
	- Mandate and autonomy of the purchasers, and purchasing power of the purchasing agency
	- Provider autonomy and Public financial management rules
	- Information system architecture
	- HMIS capacity
The health care goods and services to purchase	- Benefits package
	- Service delivery standards, including gate-keeping and referral guidelines, and clinical guidelines
	- Medicines and prescribing guidelines
The providers from whom goods and services are purchased	- Rules for selective contracting
	- Private sector engagement
How to purchase: contracting and provider payment	- Contracting
	- Provider payment
	- Provider monitoring

### Study design

The study adopted a convergent parallel mixed methods design involving semi-structured key informant qualitative interviews, qualitative document review and quantitative document (annual budgets) review. Documents were retrieved and reviewed at the same time as the key informant interviews were being conducted, and findings from both data collection methods were merged.

### Data collection method

#### Document search

Government documents were retrieved electronically from organizational websites including Abia State Ministry of Health, Abia State Health Insurance Agency, and Abia State Primary Healthcare Development Agency. In order to ensure that relevant documents that capture the topic of concern were not missed, the online searches were performed using various combinations of keywords and additional documents were identified from a careful study of the bibliography of already retrieved files. The keywords that were used to perform electronic search include ‘strategic health purchasing’, ‘health financing mechanisms’, ‘Abia State’, ‘health budget’, ‘social health insurance’, ‘operational guideline’, ‘operational manual’. Print copies of paper-only files were retrieved from the relevant government agencies and departments. The document search was performed by three reviewers (core review team) between August and September of 2019.

#### Data extraction

Data was extracted from each document using a uniform template co-developed by SPARC that captured specific themes and questions as outlined in Table 1. The template was prepared in Microsoft Excel. Retrieved sources were critically read to identify and document significant findings pertaining to governance arrangements and information architecture, and purchasing functions. Relevant information from each document were carefully summarized/paraphrased and entered into the excel file. Data extraction was also performed by the same reviewers who retrieved the documents.

A list of documents reviewed is attached as supplementary file 1.

#### Key informant interviews

Interviews were conducted simultaneously with the document reviews. Seven key personnel of the State Ministry of Health and the State Health Insurance Agency to gain experiential and contextual information on how health purchasing is implemented for the state budgetary allocation for health and what is proposed in the State Social Health Insurance Scheme. Participants were purposively selected. Only those who were directly involved in health purchasing were approached to be interviewed. The interviews were conducted by qualitative researchers using a semi-structured interview guide that was developed by SPARC and the technical partners. The interview guide was structured according to the themes in the document review. Participants were interviewed in their offices and each interview lasted approximately 45 min. Audio recordings of interviews were transcribed and relevant data were extracted from transcripts into a uniform template.

#### Data synthesis and analysis

Descriptive statistics was performed for quantitative data. The annual budgetary allocation to health was calculated as a percentage of the total budget.

Qualitative data was analyzed according to the pre-defined themes that were derived from the SHP progress tracking framework (Table 1) and narrative synthesis was of findings was done in stages. At the first stage, each reviewer synthesized their findings across documents and interviews for each review question, citing all the sources from which specific pieces of information were retrieved. At the second stage, the core review team met to aggregate all the findings from the reviewers on each topic/review question. Having confirmed that all findings had been captured, the findings were synthesized by merging similar results, removing duplicates and drawing linkages across review questions and themes. The comprehensive excel file was shared with the wider research team for an internal peer review. Gaps in information and inconsistencies in information provided were highlighted. The core review team revised the excel file in line with reviewers’ comments and the revised file was shared with external reviewers from SPARC. Reviewers’ comments were once again used to revise the excel file which formed the basis for the results that are presented in this manuscript.

## Results

Findings on governance arrangements and information architecture, and purchasing functions are presented in detail for the GTR and the SSHIS. The pillars include governance arrangements and information architecture, healthcare goods and services to purchase, the providers from whom goods and services are purchased, and provider payment and contracting. Tables 2, 3, 4 and 5 additionally highlight what exists and what does not exist to support SHP functions in the Abia State budgetary allocation for health and the State Social Health Insurance Scheme for each SHP pillar.

**Table 2:** Strengths and weaknesses of Governance & Information infrastructure for SHP

State budgetary allocation for health	State Social health Insurance Scheme
**Strengths** include existence of - Abia SMOH as the primary purchasing agency with the mandate and autonomy to ensure standard of care - Purchasing function is primarily governed by the approved state budget and public procurement law - Unified information system (DHIS 2) and data governance structure	**Strengths** include existence of - Legal framework that established the purchasing agency (Abia State House of Assembly law 2017, vide no. 2) - Governing board that oversees and regulates the activities of the agency - Client complaints portal for gathering grievances/appeals - Quarterly performance monitoring of key indicators by the M&E unit of the agency
**Weaknesses** include - Weak capacity to implement strategic purchasing - Operation of zero and incremental budget - Weak accountability structure - Lack of implementation of sanctions/penalties for poor accountability - Lack of IT infrastructure and limited IT skills	**Weaknesses** include - Lack of information technology (IT) infrastructure and staff with relevant IT skills - Lower thresholds for accreditation of public providers who are the majority - Possibility of incomplete release of equity funds by state government
**Opportunities**	**Opportunities**
- Capacity strengthening for strategic purchasing to improve linkage of budget to operational plans and achievement of health system goals - Properly defined governance structure for strategic purchasing with clear oversight and accountability	- Maximize purchasing power by employing this single pool - Develop IT infrastructure and ensure integration with state level IT architecture
**Threat** - Rigid budget reduces the opportunity for efficiency and more strategic purchasing	**Threats** - Inadequate data-driven decision-making processes due to manual processes - Distrust by the provider population due to the application of different standards for different sectors - Changes in political landscape leading to delays in disbursement of funds

**Table 3:** Strengths and weaknesses in the SHP function of goods and services to purchase

State budgetary allocation for health	State Social Health Insurance Scheme
**Strengths** include existence of - Explicit minimum service package which was revised in 2018 - Essential medicines list and generics-only policy - Referral guidelines - Service delivery and quality standards **Weaknesses** include lack of - Implementation of gate-keeping - Transparency to clients about entitlements and obligations - Adherence to generics-only policy by health facilities	**Strengths** include existence of - Explicit minimum service package which was revised in 2018 - Essential medicines list - Mechanisms for systematic periodic review of MSP using evidence of disease burden - Referral guidelines and gate-keeping policies - Standard treatment guidelines and standards for the quality of care delivered **Weaknesses** include lack of - Incentives or disincentives to clients for compliance or non-compliance with the gate-keeping policy - Incentives or disincentives to service providers for compliance or non-compliance with the referral guideline
**Opportunities**	**Opportunities**
Work with the SHIS to align on an evidence-informed and participatory process for review of the service package Leverage already existing communication channels to inform beneficiaries of their entitlements and obligations	Align evidence-informed and participatory process for review of the service package with the MOH process
**Threats**	**Threats**
Direct access to higher level care and no adherence to generics-only policy can lead to unproductive cost escalation	Direct access to higher level care can lead to cost escalation resulting in financial unsustainability

**Table 4:** Strengths and weaknesses in the SHP function of providers from whom goods and services are purchased

State budgetary allocation for health	State social health insurance scheme
**Strengths** include - Supply side subsidies to public providers to enhance service delivery standards - Contracting with private providers for the supply of medicines, medical devices and supplies - Supportive supervision of public providers to ensure compliance to quality standards - Occasional sanctions to erring providers **Weaknesses** include - Lack of standards and performance criteria to hold providers accountable for service delivery - Lack of providers’ compliance to procuring medicines, medical devices and supplies from accredited vendors - Weak data systems to support monitoring of provider performance. - Poor coordination across levels of care	**Strengths** include existence of - Accreditation guidelines that determine eligibility for providers of healthcare services to participate for each level of care - Eligibility standards for providers of medicines, medical devices and supplies - Clear guidelines which specify that at least 30% of health service providers will be from the private sector **Weaknesses** include - Lower eligibility standards for public providers compared to private providers
**Opportunity**	**Opportunity**
Contracting with private sector providers for service provision to improve access to and quality of care	Selective contracting across public and private facilities to improve the quality of health service delivery and build provider trust
**Threat**	**Threat**
Weak data systems for performance monitoring and poor provider compliance can lead to poor quality of service delivery and undermine people’s trust in the health system	Poor quality of service delivery in public health facilities can undermine trust in the scheme

**Table 5:** Strengths and weaknesses in the SHP function of provider payment and monitoring

State budgetary allocation for health	State social health insurance scheme
**Strengths** include - Use of all public health service providers - Approved state budget used for funds allocation - More coherent PPM - Generics only policy **Weaknesses** include - Lack of criteria for provider accreditation and periodic re-accreditation - Absence of list of pre-qualified wholesale suppliers of medicines, medical devices and supplies	**Strengths** include - Performance-based criteria for provider accreditation and periodic re-accreditation - Coordinated blended provider payment mechanisms - Generics-only policy for purchasing - Fixed official tariff applicable to all service providers **Weaknesses** include - Lack of coherence of the multiple PPMs - Absence of a list of pre-qualified wholesale suppliers of medicines, medical devices and supplies
**Opportunities**	**Opportunities**
Utilize more flexible PPMs e.g. global budget to increase provider autonomy and efficiency	Institutionalize the collection and review of data from providers for performance monitoring and to inform payment rate adjustments Upgrade IT systems to support claims management and data collection for performance management
**Threats**	**Threats**
Rigid line item budget with little opportunities for efficiencies Poor quality service delivery	Cost escalation due to incoherence of multiple PPMs Manual systems with data in difficult-to-analyze formats No budget for monitoring and evaluation

### Governance arrangements and information architecture

Governance arrangements and information architecture has four sub-themes namely mandate and autonomy of purchasers, legal and regulatory environment, purchasing power of the purchaser, and information system architecture and capacity.

#### Mandate and autonomy of the purchaser

The Abia State Ministry of Health (SMOH) is the primary purchasing agent for GTR-funded system. It has the mandate and autonomy to supervise and regulate all public and private primary and secondary health facilities to ensure that a minimum standard of care is maintained in health service delivery. It also oversees the activities of other agencies within the funding scheme as well as the Social Health Insurance Scheme. The Ministerial Tenders Board which comprises all Directors and Heads of Department in the Ministry is responsible for procurement of medicines, medical products and supplies for the SMOH and related agencies. The Board has the autonomy to solicit bids and select vendors for medicines, medical products and supplies.

Abia State passed a law in January 2017 which established a mandatory health insurance scheme (Abia State Health Insurance Scheme). The proposed scheme will employ a single pool at the state level and provide coverage to all residents of the State. The Abia State Health Insurance Agency is the designated purchaser under the Scheme. ABSHIA is mandated by Abia State House of Assembly law 2017 (vide no. 2) to regulate and provide oversight of all activities and programmes under the scheme, including defining provider payment mechanisms, determining provider payment rates and outlining the service benefit package.

The agency has developed an operational guideline that specifies the health benefit package and provider payment rates for capitation and fee-for-service. The agency has also defined a set of criteria for monitoring the performance of all categories of providers for the healthcare services in the benefit package.

#### Legal and regulatory environment

The purchasing function of the SMOH is primarily governed by the approved State budget and the Abia State Public Procurement Law (2012). They are also guided by other national procurement and public finance management laws. Similarly, the procurement decisions of the Ministerial Tenders Board are guided by the public procurement policy of Nigeria. Decisions on budgeting, planning and execution are made in the Department of Planning, Research and Statistics with the approval of State Planning Commission, State House of Assembly and the Governor’s office.

ABSHIA has a governing board that oversees the activities of the agency, and administrative departments and units that manage the day to day affairs of the agency. Other governance structures include the Law that established the agency and provides legal backing to ABSHIA, and the Operational Guideline which is a secondary regulation that specifies how the scheme will be implemented in keeping with the law. The agency engages enrollees, healthcare providers, Third Party Administrators, vendors, ABSHIA staff, other ministries in Abia State, international partners, and other actors. Structures that have been put in place to ensure transparency and financial accountability of the agency include, (i) the production of an annual financial report; and (ii) a client complaint portal where beneficiaries’/clients’ can report their grievances and appeal for redress.

#### Purchasing power of the purchaser

Budgetary allocations to health were 13% in 2014, 11% in 2015, 12.9% in 2016, 9.94% in 2017, 8.7% in 2018, and 9.6% in 2019 [22]. However, health facilities do not receive funds directly from the SMOH. Staff salaries are paid directly by the state government through the State Ministry of Finance, and operational costs are covered using internally generated revenue from health facilities. The SMOH operates incremental budgeting for recurrent and capital expenditure. Once the budget ceiling is released by the State Planning Commission, a draft budget is prepared and an internal budget defense is done within the Ministry. The budget is then submitted for approval and assent as previously described. In the event of budget deficits, the Ministry applies for supplementary budget from the government through the State Planning Commission.

An initial take-off grant of ₦105 million has been approved in the 2020 State budget for ABSHIA. ABSHIA’s 2020 budget was based on the costs of health services and operational activities, with consideration of available resources. In the future, the plan is to set budget ceilings using historical data on resource utilization. The budget classification is a combination of economic and administrative classification and is an inputs-based line item budget.

#### Information system architecture and capacity

The information system architecture of the State Ministry of Health is largely unified in the DHIS 2 which is an open source software that captures routine health data and aggregates this data by facility. There are dedicated staff in the Ministry who analyze this data at the end of the month to determine facility attendance and relevant programme indicators for decision making. Data governance is managed at three levels by the Technical Working Group (TWG), the Health Data Consultative Committee (HDCC), and the Health Data Governance Committee (HDGC). The HDGC is the apex committee and comprises of representatives from all health parastatal and agencies, local government chairmen, and implementing partners.

Table 2 summarizes the strengths, weaknesses, opportunities and threats (SWOTs) of the governance structure and information architecture of the two health financing mechanisms.

### The healthcare goods and services to purchase

Findings on healthcare goods and services to purchase are discussed under four sub-themes namely, benefits package, service delivery standards, and medicines and prescribing guidelines.

#### Benefits package

The Abia State Ministry of Health has an explicit minimum service package (MSP) for GTR which was revised in 2018 for services provided in public health facilities for primary and secondary levels of care. The MSP was adapted from the national package to the prevailing health needs and the resources of the state. Services covered in primary health centers include preventive, promotive and curative care for minor ailments and injuries. It also includes maternal and child health services for uncomplicated conditions. Secondary hospitals additionally provide management of complicated malaria and complicated pregnancies, and general surgical procedures including caesarean section. User fees are paid by clients at the point of receiving care based on formal fee schedules in facilities. However, facilities are not required by law to inform citizens about their entitlements and obligations under the benefit package.

The proposed basic minimum package of health services (BMPHS) of the SSHIS was also adapted from the national MSP through a similar process as the SMOH. Services covered include basic laboratory investigations; immunization services; basic surgical procedures; treatment of common diseases (malaria, diarrheal, ENT infections, HIV); management of non-communicable diseases (hypertension, DM); maternal, newborn and child health care; eye care and emergency care; dental care; public health education and promotion; in-patient admissions (to maximum of 10 days cumulative per year. The benefit package stipulates a 10% copayment for prescribed medicines included in the medicines list (which was adapted from the generics-only national essential medicines list), and 50% copayment for highly specialized care and diagnostic tests. It is proposed that in the first five years of implementation, priority will be given to enhancing population coverage rather than benefit package expansion. Subsequently, the benefit package shall be systematically reviewed every 3 years with evidence of disease burden from DHIS through stakeholder consultation, experience sharing, validation and dissemination.

#### Service delivery standards

Purchasing decisions using the GTR are based on existing service delivery and quality standards. Service providers are assessed using relevant guidelines; clinical guidelines are used to update practicing license for health professionals; and referral guidelines exist for referring clients from primary to secondary facilities. However, there is no incentive for presentation of non-emergencies to primary care providers first, nor any disincentive for bypassing them to the next levels of care (gate-keeping).

The NHIS quality standard (checklist) was adapted to current realities in the state following a stakeholder review meeting to develop the SSHIS Operational Guideline and standard treatment guidelines. The Operational Guideline asserts that the contracts of service providers will be terminated if they fail to improve against a set scorecard or if they do not pass the periodic re-accreditation.

Although referral guidelines exist to enable gatekeeping, there are no incentives or disincentives to clients for compliance or non-compliance with the referral guidelines.

#### Medicines and prescribing guidelines

There is a generics-only policy for the purchase of medicines using the GTR, but health facilities do not adhere to the generics-only policy in their purchase of drugs from the open market. Under the State Social Health Insurance Scheme, it is proposed medicines will be purchased based on a generics-only policy. However, compliance remains to be determined when implementation commences.

The pharmacy department of the SMOH defines the medicines list based on the minimum service package for each level of care using the state budget. While the national essential medicines list has been adapted for SSHIS.

Table 3 highlights the SWOTs of both health financing mechanisms in defining the package of services to be purchased.

### The providers from whom goods and services are purchased

The providers from whom goods and services has two sub-themes namely, rules for selective contracting and private sector engagement.

#### Rules for selective contracting

The GTR-funded system does not require the purchasing agency (that is the SMOH) to enter into any contractual agreements with public providers, nor does it exclude providers who do not meet standards.

With the SSHIS, contracting with providers will be selective. However, the rules for selective contracting are different for public and private providers. The private providers will be required to meet specified standards in the Operational Guidelines to get accredited. However, all public providers will be given provisional accreditation and there will be no contractual agreement. Accredited private providers will be reassessed every three years, and based on performance their contracts will either be renewed or withdrawn. Nonetheless, within the three-year period, a contract may be suspended or withdrawn based on complaints from beneficiaries or poor performance.

#### Private sector engagement

The GTR does not contract with private providers. Whereas, in the SSHIS, it is planned that at least 30% of service providers will be selected from the private sector.

The SWOTs of both health financing mechanisms in the SHP function of providers from whom to purchase are outlined in Table 4.

### How to purchase: provider payment and monitoring

The findings on how to purchase are presented under two sub-themes, namely, provider payment and monitoring provider performance.

#### Provider payment

Within the GTR, service providers receive a monthly salary irrespective of performance or outputs. Additionally, clients make out-of-pocket payments for the health services for which user fees are charged. Out-of-pocket payments (OOPs) are made for registration (card), consultation, laboratory tests, medicines, in-patient stay/admissions, surgeries and other procedures. Conversely, SSHIS proposes to pay providers using a combination of capitation, per diem and fee-for-service (FFS), using pre-defined rates based on actuarial analysis. Capitation shall be paid a week before the capitation month for a defined set of services to be rendered to beneficiaries registered with the service provider. Per Diem reimbursements will be paid for bed space per day during hospitalization, whilst FFS reimbursements will be paid for emergencies. Primary providers will be entitled to the three payment mechanisms while secondary providers will be entitled to FFS and per diem reimbursements. For both FFS and per diem, the vetted figure shall be remitted to the provider’s account through electronic payment within 30 days of submission of claims.

#### Monitoring provider performance

There is no clearly defined path for ensuring quality of care within the GTR. Although service providers are expected to turn in periodic reports of their performance, this information is not used to modify provider payment. Unannounced supervision visits are sometimes used to identify poorly performing service providers, and “repeat offenders” who may then be subjected to sanctions such as redeployment, disciplinary panels, and very rarely withholding of salaries. It has been stated that gross indiscipline and favoritism make it difficult to implement disciplinary measures on erring service providers.

Several measures are proposed for monitoring the performance of service providers within the SSHIS and these include, (i) monthly activity reporting to the local government and ABSHIA; (ii) quarterly monitoring of key performance indicators by ABSHIA; (iii) annual quality assessment of accredited service providers; (iv) performance-based payments; and (v) client complaint portals. It is also proposed that random client surveys would be used to monitor provider behaviour, actual service utilization, and client satisfaction with quality of care. The ABSHIA Operational Guideline proposes that service providers who fail to meet the minimum performance standards shall lose their accreditation status.

The strengths and weaknesses of both health financing mechanisms with respect to the SHP pillar of provider payment and monitoring are highlighted in Table 5.

## Discussion

This study examined general trends in strategic purchasing of two health financing mechanisms with a view to highlight any gaps that may exist and identify where progress in being made that offer opportunities for learning. Our findings show that health services are purchased passively using GTR, whereas the SSHIS proposes a more strategic purchasing arrangement. The failings of the GTR-funded system are reflected in the weak governance arrangements with respect to purchaser-provider split, the lack of rules for selective contracting and engagement of private providers, and lack of performance-based PPM. However, the GTR adopts a defined benefit package, which ensures value-for-money, as the basis for resource allocation. The SSHIS, on the other hand, proposes a separation of functions of the purchasing agency and the provider, selective contracting of providers (including private providers), and performance-based PPM.

Regarding the governance arrangement of the GTR, it is based on historical budgeting systems which do not necessarily take into consideration any past outputs or performance. Furthermore, the purchasing agency (SMOH) is also involved in service provision, and this has negative implications for provider performance monitoring. The SMOH (purchasing agency) is unlikely to penalize (or delist) underperforming public health facilities which are under its jurisdiction, as this may not reflect well on the Ministry as a service provider. Hence, providers are not incentivized to perform better. Moreover, provider payments (monthly salaries) are not linked to performance. and health facilities do not receive funds for operational costs from the State Ministry of Health. Hence, there are no incentives for providers to improve quality or standard of care or increase outputs (in terms of number of clients served).

Moreover, health financing in the state is constrained by weak institutional structure and policy environment for health financing; low government health spending; very high levels of out-of-pocket spending; low level of coverage of health insurance and other pre-payment and financial risk protection mechanisms; poor resource mobilization; allocative inefficiency and corruption [18]. User fees that are used to supplement the meagre budgetary allocation to health and usually paid through OOPS by clients at the point of receiving healthcare has reduced the affordability of care for many and promoted late presentation of cases, with its associated complications. Similar experiences were reported in China where rapid cost increases were preceded by reorganization of health financing mechanisms [25]. Although salaries do not incentivize provider productivity, it is unlikely that government workers will accept an output based payment system, because of potential problems with labor unions.

In order to make purchasing through annual budgets to become strategic and improve quality of services, information on provider performance should be collected regularly, analyzed and used to address areas of poor performance. Although failure to do this has been attributed to lack of funds, this can be addressed by including this activity in the line budget of the State Ministry of Health. A strong data system will be required to support monitoring of provider performance, with well outlined parameters to be monitored, if good results are to be expected. There should also be regular analysis of this data with capacity to interpret it and make changes in the methods, when necessary. The use of effective claims management software and electronic patient registers will help improve provider performance monitoring systems.

The purchasing mechanisms in the proposed Social Health Insurance Scheme represents a major opportunity for boosting implementation of SHP in the state. The establishment of a purchasing agency whose functions and operations are backed by a legal framework would ensure some level of sustainability of the scheme and provide authority for the agency to implement its Operational Guideline. Furthermore, the involvement of both private and public providers will increase access to care, create room for healthy competition as well as coordination between public and private providers in which cross-referrals are incentivized, and improvements are made in continuity and quality of care [26, 27].

The State Social Health Insurance Scheme proposes to use multiple and mixed provider payment mechanisms including capitation and fee-for-service for primary care, fee-for-service for secondary care and per-diem for hospitalization. Varying degrees of success have been recorded by different countries with the use of different payment mechanisms [28, 29], and although most forward-looking health financing mechanisms adopt blended mechanisms for payment of providers, mixed PPM could be incoherent. Providers are incentivized to shift clients, services and/or resources from less profitable to more profitable payment mechanisms [30–32]. FFS reimbursements incentivize providers to increase the number of services delivered, above what is medically necessary while per-diem could incentivize providers to raise the number of hospital admissions (including unnecessary admissions) and increase the length of hospital stay for in-patients [31, 33]. These behaviors result in cost escalation and resource wastage that jeopardize potentials for SHP to contribute to efficient utilization of limited resources to provide quality health services for those in need. In order to minimize this, gate-keeping should be incentivized, public cost of admissions across hospitals should be transparent, fee schedules should be periodically reviewed, and providers should be monitored strictly to improve provider performance.

In addition to the previously discussed potential threats with PPMs, there are a few issues that could (and indeed would) challenge sustainable implementation of a social health insurance scheme in Abia State. The challenges to implementation including, i) lack of skills and equipment for electronic claims management which could hinder provider performance monitoring; ii) poor state of infrastructure and chronic shortage of critical health workforce in primary health centers which comprise majority of primary care providers in the scheme, could demotivate participation in the scheme and renewal of registration by beneficiaries.

In order to make purchasing functions in the State Social Health Insurance Scheme more strategic, the purchasing agency needs to build or strengthen staff capacity for SHP and equip them with the relevant IT infrastructure to manage claims and track provider payments. They should strengthen the gate-keeping policy by specifying and implementing incentives or disincentives to clients for compliance or non-compliance with referral guidelines. They should apply the same selection criteria (standard of care requirement) for all providers regardless of type (public providers should have the same standards as private providers). Moreover, institutionalization of SHP in Abia State and the rest of Nigeria will require strengthening the capacity of purchasing agencies and raising awareness of its benefits amongst decision makers in the Ministry of Finance and various departments, agencies and programmes at the Ministry of Health at the federal and state levels.

This study has some limitations, whereas we assess the current functioning of the GTR, the SSHIS has not started implementation. The current design may seem to overcome many shortcomings of GTR but only after implementation can we assess if these design objectives have been met and improvements in defining benefits packages, contracting, provider payment and monitoring provider performance are observed. Street level bureaucrats such as service providers have been noted to change the implementation of policy based on their understanding of the policy objectives or to meet their own goals. Further this assessment is a cross-sectional view of the health system but will require updating over time to be able to track changes in the performance of purchasing particularly for SSHIS which is yet to be initiated. Despite these limitations, this study provides important lessons for other States in Nigeria considering establishing a SSHIS to expand coverage. This study provides considerations for the design of future SSHIS to consider where there are overlaps, duplications and gaps to strengthen in how benefits are designed, how providers are contracted and paid.

## Conclusion

In conclusion, the purchasing arrangement of the Abia State GTR-funded system is passive and is not designed in its current form to support the SHP system in terms of provider selection, provider-purchaser split and provider payment and contracting. The proposed purchasing functions of the SSHIS is largely strategic and in its current form is designed to counter shortcomings in GTR and support the SHP system in terms of provider selection, provider payment and contractual arrangements. Health purchasing in the two financing mechanisms can be made more strategic by strengthening the capacity of purchasing agencies to analyze, interpret and use data for decision-making. Furthermore, SHP could be institutionalized in Abia State and the rest of Nigeria by creating awareness of its benefits amongst decision makers in the Ministry of Finance and various departments, agencies and programmes at the Ministry of Health at the federal and state levels.

## Supplementary Information

**Additional file 1: Supplementary file 1.** List of official documents reviewed.

## Data Availability

The datasets generated and analyzed during the current study are available from the corresponding author on reasonable request.

## Abbreviations

ABSHIA: Abia State Health Insurance Agency
BHCPF: Basic Health Care Provision Fund
BMPHS: Basic Minimum Package of Health Services
FFS: Fee-for-service
GDP: Gross Domestic Product
GTR: General Tax Revenue
HMIS: Health Management Information Systems
LGA: Local Government Area
LMICs: Low- Middle-Income Countries
MSP: Minimum Service Package
NHIS: National Health Insurance Scheme
OOPS: Out-of-pocket spending
PHC: Primary Health Care
PPM: Provider Payment Mechanisms
SHP: Strategic Health Purchasing
SMOH: State Ministry of Health
SPARC: Strategic Purchasing Africa Resource Centre
SSHIS: State Social Health Insurance Scheme
SWOT: Strengths, Weaknesses, Opportunities and Threats
THE: Total Health Expenditure
UHC: Universal Health Coverage
