# Supplementary material for: Supporting strategic health purchasing: a case study of annual health budgets from general tax revenue and social health insurance in Abia state, Nigeria
Source: Health Econ Rev. 2021 Dec 20;11:47. doi: 10.1186/s13561-021-00346-8 (PMC8690461; doi:10.1186/s13561-021-00346-8)
Supplement: Supplementary file 1 — Additional file 1: Supplementary file 1. List of official documents reviewed. [file 13561_2021_346_MOESM1_ESM.docx]

**SUPPLEMENTARY FILE 1: List of official documents reviewed**

| 1. Health sector financing landscape in Abia State: Findings of Performance Evaluation Review, Fiscal Space Analysis and HHES |
| --- |
| 1. Abia State Health Insurance Agency. Summary Report of Assumptions for Actuarial Costing of Abia State Social Health Insurance Scheme Benefit Package |
| 1. Abia State Health Budget Analysis (2014-2019) |
| 1. Operational Guideline (Draft) of the Abia State Health Insurance Scheme |
| 1. Consolidated Provider Monitoring Plan (PMP) Template for the Abia State Health Insurance Agency |
| 1. Actuarial Analysis and Costing of benefit package for the Abia State Health Insurance Scheme |
| 1. Abia State Health Insurance Agency. Professional Service Charges |
| 1. Abia State Health Insurance Agency. Provider Payment Mechanisms (Draft) |
| 1. Abia State Health Insurance Agency. Health Benefit Packages Scenarios with Mini Package |
| 1. Abia State Health Insurance Agency. Financial Management Guideline (Draft) |
| 1. Abia State Combined PER & Fiscal Space Analysis Workbook. (Recent and Updated) August 2019 |
| 1. Abia Fiscal Space Analysis Scenarios – 1^st^ draft |
| 1. Abia BHCPF Training and Operational Update. PowerPoint Presentation, January 2020 |
